# Efficacy and Safety of a Permethrin-Fipronil Spot-On Solution (Effitix®) in Dogs Naturally Infested by Ticks in Europe

**DOI:** 10.1155/2016/9498604

**Published:** 2016-09-15

**Authors:** Christelle Navarro, Nadège Reymond, Nolwenn Crastes, Stéphane Bonneau

**Affiliations:** ^1^Virbac, 13ème rue, LID, 06511 Carros, France; ^2^Nadege Savelli EIRL, 2 rue de l'Église, 06270 Villeneuve-Loubet, France

## Abstract

Effitix is a new broad spectrum product based on the combination of fipronil 6.1% and permethrin 54.5% in a solution for spot-on application. It has been shown to be safe and efficacious in dogs in controlling tick, flea, sandfly, and mosquito infestations in laboratory conditions. The aim of this controlled, randomised study was to assess its safety and efficacy against natural tick infestations in field conditions. One hundred eighty-two privately owned dogs were included in France and Germany: 123 dogs were treated on day 0 with the permethrin-fipronil combination (Effitix) and 59 with a permethrin-imidacloprid combination (Advantix®). Tick counts were conducted on days 0 (before treatment), 7, 14, 21, and 28. The percentages of efficacy on days 7, 14, 21, and 28 were, respectively, 91.2%, 97%, 98.3%, and 96.7% with Effitix and were 94.8%, 96.9%, 95.7%, and 94.6% with Advantix. Very few adverse events were reported. Most were not serious and/or not related to the treatment with pruritus being the most common. One administration of Effitix was highly effective and safe to treat and control tick infestations for four weeks in field conditions and had a similar efficacy as the permethrin-imidacloprid combination for all visits.

## 1. Introduction

Vector-borne diseases include a group of globally distributed and rapidly spreading illnesses that are caused by a range of pathogens (bacteria, viruses, protozoa, and helminths) transmitted by arthropods, in particular ticks, fleas, mosquitoes, and phlebotomine sandflies [1–4]. Controlling the vectors is important because they are responsible for the transmission of serious disease in dogs such as babesiosis, canine leishmaniosis, dirofilariosis, and Lyme borreliosis. In addition to their veterinary importance, some canine vector-borne diseases are of major zoonotic concern. The explosion of canine populations and their close relationship with humans poses new concerns for human public health, dogs being competent reservoir hosts of several zoonotic agents [5]. It is now widely accepted within the scientific community that veterinarians, physicians, and pet owners must take measures to protect pets from parasite infestations. This protection is needed not only to relieve the pets from the mechanical irritation and inflammation caused by the parasites but also to protect them from the potential vector-borne diseases they may carry and even more importantly to limit the risks associated with zoonotic transmission of parasitic diseases. Ectoparasite management is a perfect example of the “one health” approach [3, 6]. Current recommended prevention strategies include the application of acaricides, insecticides, and repellents directly on the dog, in the form of collars and spot-on and spray formulations [2]. By rapid killing and/or by preventing the parasites from taking a blood meal, they reduce the risk of canine vector-borne disease transmission. The choice of treatment should be adapted to the local epidemiological situation. However, as a result of the changes in distribution of vectors, along with increasing frequency of pet travel, it may also be vital to consider protecting pets from as many vectors as possible and to maintain the year-round protection. This applies even in areas where some of these vectors are not traditionally endemic [7]. Effitix is a new broad spectrum product recently authorised to be marketed in Europe. Effitix is a combination of two active ingredients, fipronil 6.1% and permethrin 54.5%, in a solution for spot-on application. Fipronil has a well-established efficacy to control flea and tick infestations and is widely recommended ingredient in veterinary medicine since its first registration as spray formulation in 1994 [8, 9]. Permethrin has strong repellent effects, in particular against Diptera. These effects are sufficient to cause disorientation and irritation resulting in the absence or reduction of blood feeding (antifeeding effect) [10–14]. Permethrin can also act as an acaricide and insecticide. The spectrum of activity of permethrin includes flies, mosquitoes, fleas, ticks, lice, and mites [15]. The results presented below are from one field efficacy study performed in Europe to confirm the acaricidal efficacy of the combination in dogs naturally infested by ticks.

## 2. Materials and Methods

### 2.1. Animals, Products, and Protocol

The study was conducted between March and December 2012 as a multicentre, positive controlled, blinded, and randomised clinical trial. It was carried out in 33 veterinary practices at different locations in France and Germany incorporating a total of 182 privately owned dogs in which at least three live attached ticks had been diagnosed. Inclusion was based on the following criteria: age ≥ 12 weeks; body weight ≥ 1.5 kg; not living with more than three dogs in the household; healthy or disease under control; signed owner consent and owner's agreement to attend the visits planned in the protocol. Dogs presenting with the following were not included in the study: pregnant or lactating females or those intended for breeding during the next four weeks; dog or its environment treated with a parasiticide with ongoing tick efficacy as per label; major surgery within seven days prior to inclusion or planned during the study period; severe chronic disease; requirement for a concomitant treatment which was not allowed by the protocol; requirement for a shampoo within the first 2 weeks of the study; dog known to have had hypersensitivity to a spot-on solution or to one of the ingredients of the products tested. Forbidden concomitant treatments were any ectoparasiticides other than the investigational products and any environmental product with efficacy against ticks and/or fleas. At visit V1, on day 0, each dog received a single treatment according to the randomisation plan. Dogs were treated with a permethrin-fipronil spot-on (Effitix, Virbac) or with a permethrin-imidacloprid spot-on (Advantix spot-on, Bayer) solution. The randomisation was performed by an algorithm implemented on Vision® 7 for each study site. One randomisation list common for single and multidog households was given to each site. Animals were randomised block-wise in order of presentation to the site using sets of three households with a 2 : 1 (Effitix : Advantix) enrolment ratio. The size of the pipette of the product to apply was chosen according to the body weight of the dog and following the label recommendations. The five presentations of Effitix spot-on solution (i.e., 0.4 mL, 1.1 mL, 2.2 mL, 4.4 mL, and 6.6 mL) with 61 mg of fipronil and 545 mg of permethrin per mL were tested versus the four presentations of Advantix spot-on solution (i.e., 0.4 mL, 1.0 mL, 2.5 mL, and 4.0 mL) containing 100 mg of imidacloprid and 500 mg of permethrin per mL. Since the Effitix and Advantix spot-on solution had different appearances, blinding of veterinary assessments was ensured by the “blinding by function” technique: a clinician (veterinary investigator) was responsible for efficacy and tolerability evaluations and a dispenser was responsible for treatment delivery and compliance. Dispensers were veterinarians, veterinary nurses, or suitably qualified personnel. The study was conducted in compliance with Good Clinical Practice [16] and with the guidelines for the testing and evaluation of the efficacy of antiparasitic substances for the treatment and prevention of tick and flea infestation in dogs and cats [17]. The study was performed in accordance with the national ethical rules and all the pet owners gave their consent for the participation to the study.

### 2.2. Tick Count

An initial parasite count was performed followed by administration of the treatment in the veterinary practice. A tick count was then performed at each visit. Tick counting was performed by visual inspection and palpation and by systematically pushing the hair against the hair growth so that ticks became visible. The attached ticks were then removed and counted and recorded as alive or dead. The entire dog was examined continually until five minutes after the last tick was found. A minimum examination time of five minutes was required. The ticks were then placed into containers with alcohol (one for live ticks, one for dead ticks) and sent to a laboratory for identification. A microscopic identification was performed and the tick species and stage of development were recorded on the Tick Species Identification Form. If the number of ticks per sample exceeded the number of 30 ticks, the laboratory sorted the ticks first according to the genus (*Ixodes*,* Dermacentor*,* Rhipicephalus*, and others) and then performed a species identification in a maximum of 30 randomly selected ticks per genus. The type of* Rhipicephalus *was not identified.

### 2.3. Monitoring and Recording Adverse Events

A full physical examination, including measurement of the rectal temperature, abnormal signs since last visit, and application site abnormality, was performed at each visit. Any abnormalities which were not present before the treatment were recorded as adverse events and were evaluated with regard to their seriousness and potential relationship with the treatment. The causality assessment was evaluated using the ABON system (A = Probable, B = Possible, O = Unclassifiable, and N = unlikely to be product related) [18, 19]. The ABON classification was performed by blinded personnel. Due to the high sensitivity of cats to permethrin, the investigator recorded any abnormal clinical signs on cat(s) which they considered to be related to the administration of permethrin to the dog(s) living with the cat(s) (neurological, gastrointestinal, respiratory, and/or abnormal behaviour signs).

### 2.4. Criterion of Efficacy and Statistical Analysis

The statistical analysis was performed using validated statistical programs with the software SAS® version 9.2. The homogeneity of the two groups was analysed using the following parameters: age, body weight, gender, breed, reproductive status (intact/neutered), hair coat (short/medium/long), sleeping place (inside/outside), number of dogs in household, number of cats in household, and medical history (yes/no). The homogeneity of the two groups was also analysed for the live tick count at day 0. To test homogeneity between arms a Wilcoxon test was performed on continuous data (age, body weight, number of dogs in household and number of cats in household, and live tick count at day 0) and a Fisher's test was used on discrete data (other parameters).

The primary criterion was the percentage reduction of the live tick count between the baseline (D0) and the postbaseline (D7, D14, D21, and D28) visits.

The percentage reduction of the live tick count was calculated for each dog as described:

 Reduction of live ticks (%) = 100 × (number of live ticks on D0 − number of live ticks on D7, D14, D21, or D28)/number of live ticks on D0.

95% confidence intervals of intertreatment difference were calculated with the bootstrap method for the efficacy percentages' arithmetic and geometric means. A noninferiority of Effitix in relation to Advantix could be concluded if the lower bound of the 95% confidence interval of the difference between groups was greater than −10%.

## 3. Results

One hundred and eighty-two dogs were included from 20 March 2012 to 16 November 2012. The last visit was performed on 15 December 2012. Among the 182 dogs included in the study, 181 dogs were classified in the Intention to Treat population, 170 dogs were classified in the Per Protocol population, and 181 dogs were classified in the Safety Set population. Out of 181 dogs, 123 were treated with Effitix and 58 with Advantix. Given the high similarity between the Intention to Treat and the Per Protocol analysis, the efficacy results are given for the Intention to Treat analysis only.

### 3.1. Site Distribution

**Figure 1:**
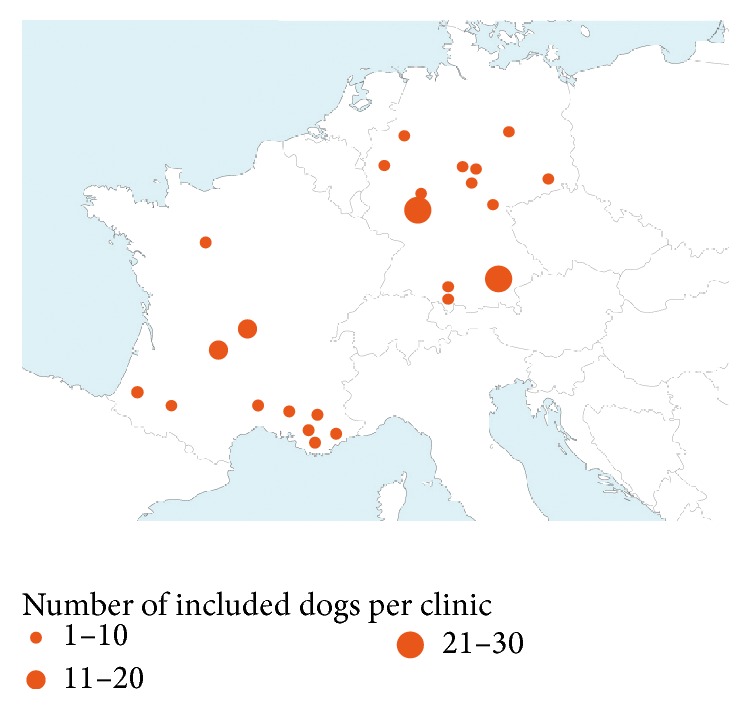
Site distribution: location of participating practices in France and Germany.

A total of 182 cases were recruited, 85 in France (between 1 and 19 dogs per site) and 97 in Germany (between 1 and 29 dogs per site). No site enrolled more than 40% of the evaluable cases. The number of cases enrolled by each site and their location are given in Figure 1.

### 3.2. Homogeneity at Baseline

Analyses of demographic data confirmed that case allocations between treatment groups were balanced. There was no difference between groups at baseline concerning age, body weight, sex, gender, reproductive status, breed distribution, hair coat length, medical history, number of dogs in the household, number of cats in the household, and the live tick count on day 0. The results are summarised in Tables 1 and 2.

### 3.3. Tick Identification

The number of live ticks collected before treatment ranged between three and 735. All tick species commonly encountered in Europe were represented in the study. As regards adult tick species, the most widespread species in the study on day 0 were* Rhipicephalus *spp. The next most frequent species were* Ixodes hexagonus* and* Ixodes ricinus*. The least frequently found species was* Dermacentor reticulatus*.

Table 3 reports the tick species and stage of development identified.

### 3.4. Efficacy Assessment

#### 3.4.1. Percentage of Reduction of Live Tick Count

**Figure 2:**
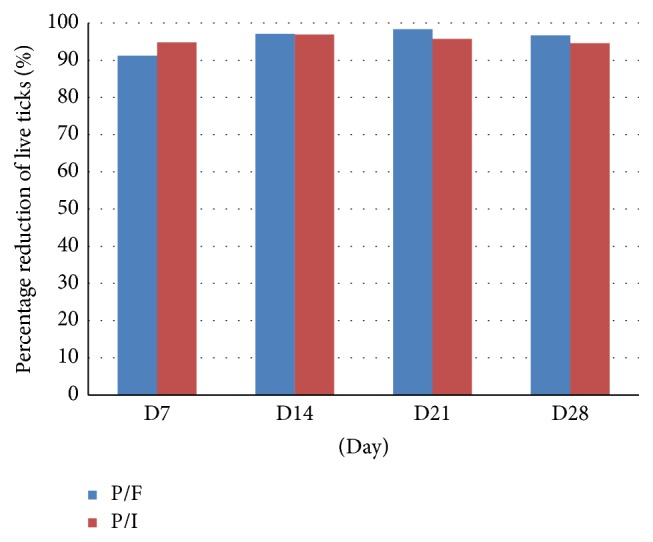
Percentage of reduction of live ticks (geometric means) (P/F = permethrin-fipronil spot-on solution (Effitix), P/I = permethrin-imidacloprid spot-on solution (Advantix)).

The percentages of efficacy of the Effitix group were 91.8%, 95.0%, 97.5%, and 96.7% on days 7, 14, 21, and 28, respectively, based on the Intention to Treat population and the arithmetic means and were 91.2%, 97.1%, 98.3%, and 96.7% on days 7, 14, 21, and 28, respectively, based on the Intention to Treat population and the geometric means. The percentages of efficacy of the Advantix group were 95.9%, 97.4%, 96.7%, and 92.1% on the same assessment days (arithmetic means) and 94.8%, 96.9%, 95.7%, and 94.6% (geometric means). The results based on the geometric means are shown in Figure 2.

#### 3.4.2. Assessment of the Noninferiority

Whatever the calculation method (arithmetic or geometric), the lower limit of the confidence interval was greater than −10%, so noninferiority was demonstrated at each visit for the Intention to Treat and the Per Protocol populations. According to the principle of* a priori* ordered hypotheses and as all visits showed conclusive noninferiority, we can conclude to a similar efficacy of Effitix versus Advantix at all visits. The values of the 95% confidence intervals of intertreatment difference for the Intention to Treat population are given in Table 4.

### 3.5. Adverse Events

A total of 31 dogs presented an adverse event: 17 dogs (13.8%) in the Effitix group and 14 dogs (24.1%) in the Advantix group. Some of these dogs displayed several events leading to a total of 39 adverse events: 22 in the Effitix group and 17 in the Advantix group. Among the 39 adverse events, nine were analysed as Probable, five being in the Effitix group and four in the Advantix group, ten as Possible, six being in the Effitix group and four in the Advantix group, and two in the Advantix group as Unclassifiable. The 18 remaining disorders were classified “*N*” as unlikely because an alternative explanation was given or there was a long delay between the application of the product and appearance of the events. The most widespread adverse event was “skin and appendages disorders” (eight dogs in the Effitix group (6.5%) and seven in the Advantix group (12.1%)) and consisted essentially of pruritus (seven dogs in the Effitix group and five dogs in the Advantix group).

During the follow-up visits, the veterinarians observed cases with application site abnormalities only in the Advantix group (6.9% at D7 and 3.6% at D14). These site abnormalities were oily hair, alopecia, crusts, pruritus, and inflammation. In the Effitix group, even if no application site abnormality was observed during a follow-up visit, 3 cases were notified as adverse events.

No cats living with the dogs treated presented any physical abnormality.

## 4. Discussion

The principal findings of this study are that Effitix was highly effective and well tolerated for the treatment of dogs naturally infested by ticks when administered once monthly. Effitix had similar efficacy compared to Advantix. The choice of Advantix as the positive control drug in this study was based on several considerations. Firstly, Advantix, an association of permethrin and imidacloprid, has a spot-on formulation similar to Effitix which insures better blinding for the veterinary investigator. Secondly, the study was conducted in two very different geographical areas: France and Germany, where the products are both marketed. As Effitix is active against ticks, fleas, mosquitoes, and sandflies, it was considered as relevant to include within the field trial a geographical area where the target parasites are present concomitantly. This is actually the case in the south of France where fleas, ticks, and* Phlebotomus perniciosus *are endemic [20–23]. Due to the presence of the transmitting vector, this area is also endemic for leishmaniosis [24–26]. Given the importance of leishmaniosis as a canine vector-borne disease and zoonosis, a protection against the transmitting vector* P. perniciosus* was therefore ethically considered as mandatory. Thirdly, taking into consideration the need for a broad spectrum of action covering fleas, ticks, and sandflies, Advantix was the most recent authorised product in Europe registered with a repellent (antifeeding) activity against sandflies* (P. perniciosus) *at the time of the study.

The study was conducted at a time which included the usual tick season in France and Germany. The two different countries selected represent two different geographical areas of Europe with different husbandry practices and different environmental and climatic conditions. All tick species commonly encountered in Europe were represented in the study:* Rhipicephalus* spp.,* Ixodes hexagonus*,* Ixodes ricinus,* and* Dermacentor reticulatus*.

However, since identification of* Rhipicephalus *species can be difficult, it was decided not to name the species. Indeed, although* R. sanguineus* is the predominant species from this group found in Europe and other parts of the world [27, 28], there are now doubts that ticks identified as such are really from this species. Morphological distinction between some species of* Rhipicephalus* can, in fact, be difficult and controversial [27–30]. Therefore, in order to avoid any misidentification and because identifying the species was not the primary goal of this study, we avoided naming the* Rhipicephalus* species. A precise identification of* Rhipicephalus* species could have been possible by combining morphological and molecular approaches [28, 30].

A concern of veterinarians in clinical practice is for the patient's safety when using newly registered products. The data from the field study confirm the good tolerance of Effitix in dogs as very few adverse events occurred. In addition, many of the adverse events reported can be confidently ascribed to factors not related to the treatment, such as other diseases. Among the suspected adverse reactions which can be linked to the treatment, transient cutaneous reactions at the application site (pruritus, erythema) and general pruritus were reported after use in some dogs with fewer dogs being affected by the permethrin-fipronil combination than by the permethrin-imidacloprid combination. Due to the unique physiology of cats which are unable to metabolise certain compounds including permethrin, Effitix is poisonous to cats. Specific attention was therefore paid to cats sharing the same household as the treated dogs. No signs which could be considered related to the exposure to permethrin were reported in cats living with the included dogs. This study consequently confirms that Effitix can be safely used in dogs sharing the same household as cats when label recommendations are respected.

## 5. Conclusions

One administration of Effitix was highly effective in treating and controlling tick infestations for four weeks in field conditions for all common European ticks identified during the study (*Rhipicephalus *spp.,* Ixodes hexagonus*,* Ixodes ricinus,* and* Dermacentor reticulatus*). Effitix had a similar efficacy compared to a control product (Advantix) at all visits. Moreover, Effitix was very well tolerated by the treated dogs and cats sharing the same household. Effitix offers a convenient tick control treatment for up to one month and due to the repellent activity of permethrin it can be used in areas where additional protection versus canine vector-borne disease like leishmaniosis and/or dirofilariosis is needed.

## Figures and Tables

**Table 1:** Homogeneity of demographic data at baseline.

		P/F	P/I	All
Number of dogs	*N*	123	58	181

Age (years)	Mean ± SD	5.26 ± 3.72	5.03 ± 3.63	5.18 ± 3.69
	*P* value (Wilcoxon)			0.75

Body weight	Mean ± SD	22.92 ± 12.16	23.11 ± 11.80	22.98 ± 12.01
	*P* value (Wilcoxon)			0.897

Gender	Male	59 (48.0%)	27 (46.6%)	86 (47.5%)
	Female	64 (52.0%)	31 (53.4%)	95 (52.5%)
	*P* value (Fisher)			0.875

Reproductive status	Intact	83 (67.5%)	34 (58.6%)	117 (64.6%)
	Neutered	40 (32.5%)	24 (41.4%)	64 (35.4%)
	*P* value (Fisher)			0.249

Breed	Pure breed	73 (59.3%)	34 (58.6%)	107 (59.1%)
	Mixed breed	50 (40.7%)	24 (41.4%)	74 (40.9%)
	*P* value (Fisher)			1

Sleeping place	Inside	86 (69.9%)	44 (75.9%)	130 (71.8%)
	Outside	37 (30.1%)	14 (24.1%)	51 (28.2%)
	*P* value (Fisher)			0.48

Hair coat length	Short	49 (39.8%)	27 (46.6%)	76 (42.0%)
	Medium	54 (43.9%)	22 (37.9%)	76 (42.0%)
	Long	20 (16.3%)	9 (15.5%)	29 (16.0%)
	*P* value (Fisher)			0.685

Medical history	No	114 (92.7%)	53 (91.4%)	167 (92.3%)
	Yes	9 (7.3%)	5 (8.6%)	14 (7.7%)
	*P* value (Fisher)			0.771

P/F: permethrin-fipronil spot-on (Effitix), P/I: permethrin-imidacloprid spot-on (Advantix), SD: standard deviation, and *N*: number of cases.

All *P* values were > 0.05 which implies an absence of difference between groups at baseline.

**Table 2:** Homogeneity of live tick counts on day 0 for the Intention to Treat population.

		P/F	P/I
Tick count at day 0	*N*	123	58
	Mean ± SD	20.4 ± 75.5	6.7 ± 11.3
	*P* value (Wilcoxon)		0.063

P/F: permethrin-fipronil spot-on (Effitix), P/I: permethrin-imidacloprid spot-on (Advantix), SD: standard deviation, and *N*: number of cases.

**Table 3:** Number of live ticks per dog for the Intention to Treat population on day 0.

	Adults		Larvae		Nymphs	
	*Rhipicephalus *spp.					
	P/F	P/I	P/F	P/I	P/F	P/I
*N*	21	10	0	0	3	1
Mean ± SD	57.4 ± 162.0	8.6 ± 5.7	NA	NA	21.3 ± 12.5	23.0 ± NA

	*Ixodes ricinus*					
	P/F	P/I	P/F	P/I	P/F	P/I

*N*	85	39	0	0	3	1
Mean ± SD	5.0 ± 3.3	4.2 ± 2.6	NA	NA	1.0 ± 0.0	1.0 ± NA

	*Ixodes hexagonus*					
	P/F	P/I	P/F	P/I	P/F	P/I

*N*	18	9	4	2	16	4
Mean ± SD	7.3 ± 7.9	3.1 ± 2.6	7.8 ± 6.6	8.5 ± 2.1	3.6 ± 3.7	4.8 ± 3.6

	*Dermacentor reticulatus*					
	P/F	P/I	P/F	P/I	P/F	P/I

*N*	**12**	**5**	**0**	**0**	**0**	**0**
Mean ± SD	4.3 ± 6.6	1.8 ± 1.1	NA	NA	NA	NA

	*Other*					
	P/F	P/I	P/F	P/I	P/F	P/I

*N*	1	0	0	0	0	0
Mean ± SD	1.0 ± NA	NA	NA	NA	NA	NA

NA: not applicable, P/F: permethrin-fipronil spot-on (Effitix), P/I: permethrin-imidacloprid spot-on (Advantix), SD: standard deviation, *N*: number of cases. If the number of ticks per sample exceeded 30, the laboratory sorted the ticks first according to the genus (*Ixodes*, *Dermacentor*, *Rhipicephalus*, and others) and then performed a species identification in a maximum of 30 randomly selected ticks per genus.

**Table 4:** Assessment of the noninferiority for the Intention to Treat population.

		D7	D14	D21	D28
Arithmetic mean	Efficacy % P/F	91.8%	95.0%	97.5%	96.7%
	Efficacy % P/I	95.9%	97.4%	96.7%	92.1%
	Efficacy % P/F − P/I	−4.1%	−2.4%	0.7%	4.6%
	95% bootstrap CI	[**−8.98%**; 0.48%]	[**−9.34%**; 2.42%]	[**−2.94%**; 4.60%]	[**−1.76%**; 13.41%]

Geometric mean	Efficacy % P/F	91.2%	97.1%	98.3%	96.7%
	Efficacy % P/I	94.8%	96.9%	95.7%	94.6%
	Efficacy % P/F − P/I	−3.6%	0.2%	2.6%	2.1%
	95% bootstrap CI	[**−9.62%**; 2.45%]	[**−3.27%**; 3.97%]	[**−1.09%**; 7.31%]	[**−2.48%**; 6.80%]

CI: confidence interval, P/F: permethrin-fipronil spot-on (Effitix), and P/I: permethrin-imidacloprid spot-on (Advantix).
